# Anti-Obesity Effects of LB-GABA

**DOI:** 10.3390/ijms26083554

**Published:** 2025-04-10

**Authors:** Hyein Han, Gunju Song, Jongwon Kim, Heegu Jin, Boo-Yong Lee

**Affiliations:** Department of Food Science and Biotechnology, College of Life Science, CHA University, Seongnam 13488, Republic of Korea; hyeinoo@naver.com (H.H.); juhun022188@naver.com (G.S.); nanananakim@naver.com (J.K.); heegu94@hanmail.net (H.J.)

**Keywords:** obesity, lipid metabolism, energy expenditure, 3T3-L1 cells, LB-GABA

## Abstract

Obesity is characterized by an excessive imbalance in energy metabolism and is associated with metabolic syndrome. Mammals have two types of adipose tissue: white adipose tissue (WAT) and brown adipose tissue (BAT). These are key factors in regulating the energy balance. Strategies aimed at reducing obesity should encompass not only the prevention of lipid accumulation but also the stimulation of browning in both WAT and BAT, with the aim of enhancing energy expenditure. In this study, the mechanism by which Lactobacillus brevis-fermented gamma-aminobutyric acid (LB-GABA) prevents obesity was investigated, as well as whether it induces lipolysis and browning in WAT using 3T3-L1 adipocytes. The expression of proteins involved in signaling pathways regulating lipid accumulation and degradation, as well as browning, was measured using Western blotting analysis. We demonstrated that LB-GABA significantly inhibited lipid accumulation by suppressing adipogenesis and lipogenesis. In addition, the microscopic analysis of WAT demonstrated that LB-GABA reduced the adipocyte size and the number of lipid droplets. Moreover, Western blot analysis revealed that GABA increased lipolysis and activated the protein kinase A (PKA) signaling pathway, which promotes uncoupling protein 1 (UCP1)-mediated WAT browning. In conclusion, these results suggest that LB-GABA activates energy expenditure through lipid metabolism regulation and exerts anti-obesity effects.

## 1. Introduction

Obesity is defined as an imbalance between dietary energy intake and energy expenditure and is also associated with metabolic syndrome [1]. Obesity is a major contributor to the development of metabolic syndrome, a condition that predisposes individuals to several health complications, such as type 2 diabetes, cardiovascular disease, and nonalcoholic fatty liver disease [2]. The prevalence of obesity has increased very rapidly over the last 30 years, posing a serious threat to human health and socio-economic development [3,4]. In recent years, the epidemiology of obesity, predisposing factors, and prevention mechanisms have been the focus of an increasing number of studies [5].

White adipose tissue (WAT) is an important regulator of lipid metabolism and the energy balance [6]. Two types of adipose tissue, WAT and brown adipose tissue (BAT), have been identified as the key regulators of the energy balance in mammals [7]. Obesity is caused by excessive WAT accumulation, where excess energy is stored in the form of triglycerides (TGs) [8]. BAT, composed of adipocytes containing numerous small multifunctional fat droplets, exhibits high levels of mitochondrial biosynthesis and dissipates energy as heat because it expresses uncoupling protein 1 (UCP1) on its inner mitochondrial membrane [9]. Studies have shown that cold exposure, adrenaline stimulation, and other conditions can trigger browning, which results in a shift from WAT to brown-like (beige or light) fat cells [10].

The 3T3-L1 cell line is one of the most well-established models for the study of the differentiation of preadipocytes to mature adipocytes (adipogenesis). The 3T3-L1 cell line was utilized in the study to examine the processes of browning and obesity-related properties. This particular cell line is a well-established preadipocyte cell line developed in mouse embryos. These 3T3-L1 cells manifest an adipocyte-like phenotype when cultivated under the appropriate conditions [11].

Adipocyte differentiation and subsequent lipid accumulation in mature adipocytes occur through a well-organized process of hyperplasia and hypertrophy, respectively [12]. During preadipocyte differentiation, the expression of peroxisome proliferator-activated receptor gamma (PPARγ) and fatty acid binding protein 4 (FABP4) and the elevated expression of lipogenic factors, such as lysophosphatidic acid acyltransferase theta (LPAATθ), Lipin 1, and fatty acid synthase (FAS), lead to increased lipid accumulation and the development of obesity [13]. Lipolysis is defined as the catabolic mechanism that facilitates the breakdown of TGs stored in WAT, resulting in the release of free fatty acids (FFAs) and glycerol [14]. TG hydrolysis is the result of protein phosphorylation of protein kinase A (PKA), which in turn regulates several key lipolytic proteins. In adipocytes, these lipolytic proteins control TG lipase activity [15]. These are transported across the membrane by carnitine palmitoyl transferase 1 (CPT1) and undergo mitochondrial β-oxidation [16,17]. The major lipases involved in the lipolytic process are adipocyte triglyceride lipase (ATGL), diglyceride to monoglyceride lipase, and hormone-sensitive lipase (HSL) [18,19]. Glycerol and FFAs released from WAT are transported through the blood and penetrate into other tissues, where they distribute lipids and regulate the energy balance [18]. In order to address the issue of obesity, there exist methodologies that not only prevent fat accumulation but also promote energy expenditure by activating the browning of BAT and WAT [19]. The expression of UCP1 uncouples electron transfer in the mitochondria to generate energy as heat [20]. WAT and BAT are readily interconvertible [21]. Similarly, the browning of WAT is known to require the expression of the thermogenic protein UCP1 [22], as these phenotypic changes activate thermogenesis, promoting the browning of WAT, which may be a therapeutic approach to treat obesity [23].

LB-GABA consists of about 20% gamma-aminobutyric acid (GABA) (*w/v*) and about 80% modified food starch [24]. GABA is a non-proteinogenic amino acid that is widely found in microorganisms, animals, and plants and is naturally produced in the human organism [25,26,27]. GABA is produced from glutamate within the central nervous system and functions as a significant inhibitory neurotransmitter in the human brain [28,29]. GABA is transported and used by the transporter [30,31]. The levels of GABA, which have been shown to be significantly reduced in cases of insomnia, anxiety, depression, panic disorder, and the aging process, have been identified as a chronic disease of modern people. Consequently, the supplementation of GABA has emerged as a promising therapeutic approach aimed at mitigating symptoms such as excitement, irritation, and stress while also counteracting the physical and psychological effects of aging [32,33,34,35]. The known effects of GABA are as follows: the body’s energy balance is regulated by the inhibition of the activation of these neurons through the release of the neurotransmitter GABA, and GABA has been reported to have a variety of physiological effects, including anti-inflammatory and anti-diabetic [36,37,38,39,40], in addition to its role as an endogenous inhibitory neurotransmitter in the mammalian central nervous system [35]. Due to these effects, GABA is used as an active ingredient in pharmaceuticals and food products, and its demand is increasing. However, the supply of foods containing GABA has proven inadequate in meeting this demand [41]. Consequently, GABA is predominantly produced and utilized through fermentation processes involving yeast, fungi, and bacteria [42,43,44,45]. The LB-GABA employed in this study was produced using Lactobacillus brevis fermentation, a method which we hypothesize can counterbalance the limited GABA content in food.

However, in 3T3-L1 adipocytes, it is not known whether LB-GABA acts by decreasing the expression of adipogenic proteins, such as PPARγ and FABP4; lipogenic proteins, such as LPAAT θ and Lipin 1; and FA synthesis proteins such as FAS, thereby inhibiting lipogenesis. Its ability to inhibit obesity by increasing the expression of pPKA and its downstream lipolytic proteins, increasing the expression of UCP1 proteins, and increasing the browning of white fat has not been reported. The hypothesis under investigation is that given the proven effects of GABA contained in LB-GABA on oxidative stress and associated inflammation, it would be expected to have potential benefits in terms of efficacy against obesity. In addition, the anti-obesity effect of GABA has already been revealed in previous studies [34], and it is expected that the same effect can be expected from LB-GABA, a fermented GABA. Therefore, this study focused on the effects of LB-GABA on the metabolism of 3T3-L1 cells and investigated the overall anti-obesity signaling of LB-GABA in 3T3-L1 cells.

## 2. Results

### 2.1. Effects of LB-GABA on the Viability of 3T3-L1 Adipocytes

**Figure 1:**
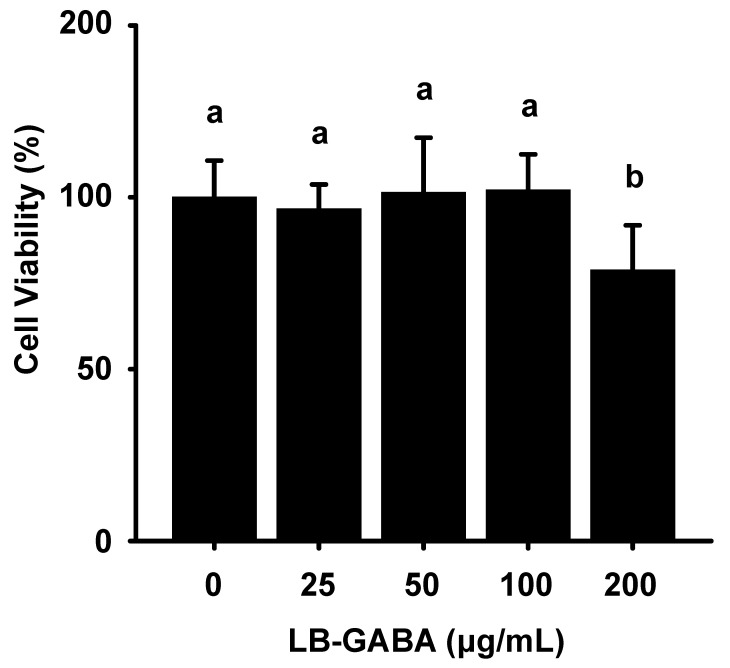
LB-GABA effects on 3T3-L1 cell cytotoxicity. The viability of 3T3-L1 preadipocytes treated with LB-GABA for 24 h was assessed using an MTT assay. Data are expressed as the mean ± SEM (*n* = 8). Values indicated by different letters are significantly different; *p* < 0.05 (a > b).

To determine the cytotoxic effect of LB-GABA on 3T3-L1 adipocytes and to determine the appropriate concentration to use in our experiments, we measured the effect of LB-GABA on lipid metabolism using the MTT (3-(4,5-dimethylthiazol-2yl)-2,5-diphenyltetrazolium bromide) assay. As shown in Figure 1 and Table S1, treatment with 200 μg/mL LB-GABA resulted in a significant difference, suggesting that it was cytotoxic; therefore, we used low and high concentrations of 25 and 100 μg/mL in the 3T3-L1 cell medium.

### 2.2. LB-GABA Inhibits Lipid Accumulation

**Figure 2:**
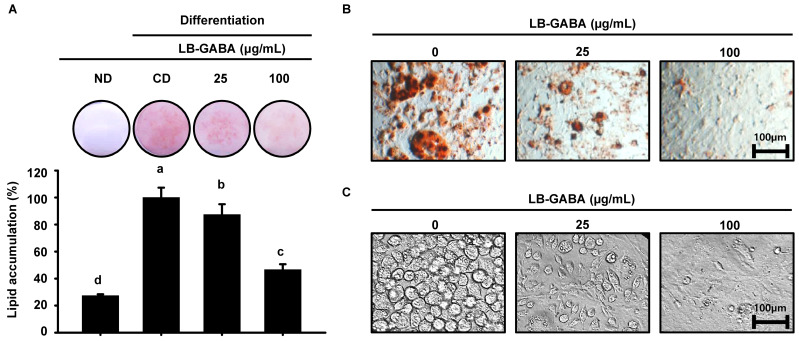
LB-GABA inhibits lipid accumulation in 3T3-L1 adipocytes. (**A**) The effect of LB-GABA on lipid accumulation, as determined by oil red O staining. A differentiation-inducing cocktail, with or without LB-GABA, was added to 3T3-L1 adipocytes. (**B**,**C**) Microscopic images of fully differentiated 3T3-L1 cells were obtained at day 8 (D8). The scale bar represents 100 μm. Data are expressed as the mean ± SEM (*n* = 12). Values indicated by different letters are significantly different; *p* < 0.05 (a > b > c > d).

To determine the effect of LB-GABA on lipid accumulation in adipocytes, we induced differentiation with IBMX (3-Isobutyl-1-methylxanthine), dexamethasone, and insulin for 8 days and treated them with two concentrations (25 and 100 μg/mL) of LB-GABA and then used oil red O staining to determine the level of lipid accumulation. As shown in Figure 2A and Table S2, differentiation induced intracellular lipid accumulation, but LB-GABA inhibited lipid accumulation in a dose-dependent manner. Microscopic examination showed that the MDI-treated group had larger lipid droplets, but the LB-GABA-treated group had smaller lipid droplets and fewer number of droplets (Figure 2B,C). Consequently, the findings of this study demonstrate that LB-GABA exerts a regulatory function on adipose tissue accumulation by reducing the magnitude and quantity of adipose tissue droplets in differentiated 3T3-L1 cells.

**Figure 3:**
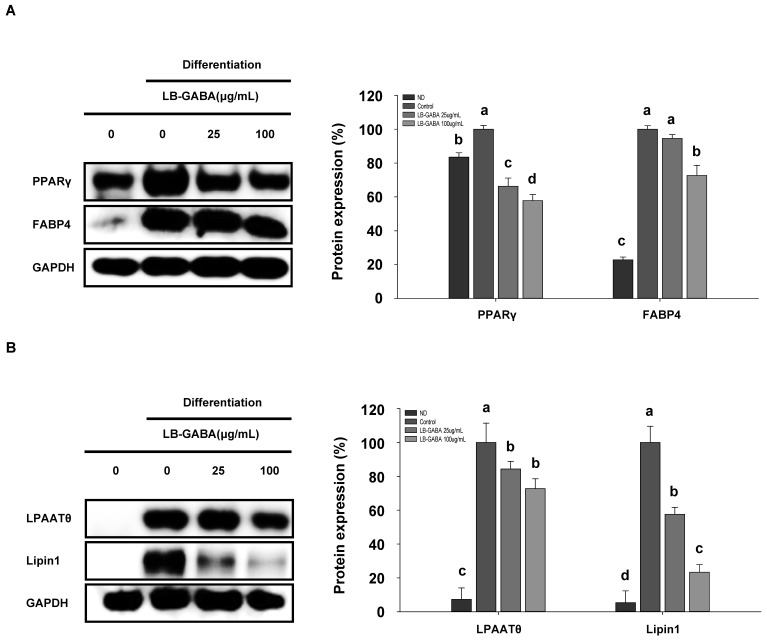

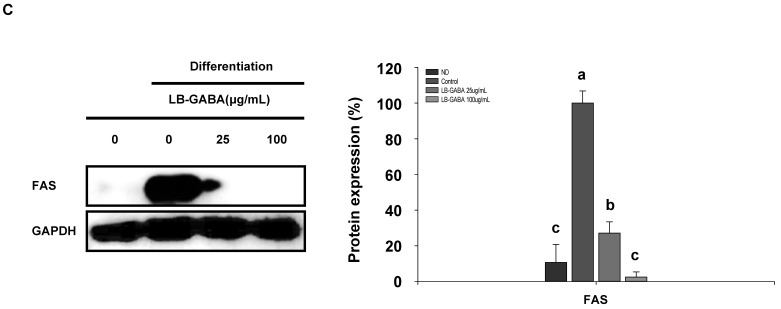
LB-GABA reduces adipogenic and lipogenic proteins in 3T3-L1 cells. Cells were incubated with various concentrations of LB-GABA for 8 days. The expression of (**A**) adipogenic proteins (PPARγ and FABP4), (**B**) lipogenic proteins (LPAAT θ and Lipin 1), and (**C**) proteins involved in fatty acid synthesis (FAS) was determined via Western blot. ND, undifferentiated; control, differentiated. Data are expressed as the mean ± SEM. Values indicated by different letters are significantly different; *p* < 0.05 (a > b > c > d).

In order to investigate the mechanism for the lipid accumulation inhibitory effect of LB-GABA, the expression of key transcription factors and biomarkers of adipocyte differentiation was measured by means of Western blot analysis. As shown in Figure 3A, Tables S3–S5 and S9–S11, the expression of key adipogenesis proteins (PPARγ and FABP4) was increased in the differentiation inducer group, but LB-GABA decreased the expression of these proteins. And as shown in Figure 3B,C, lipogenesis proteins (LPAATθ and Lipin1) and fatty acid synthesis (FAS) proteins were upregulated in the differentiation group, but LB-GABA downregulated their expression. These results suggest that LB-GABA inhibits adipogenesis and lipogenesis in 3T3-L1 cells.

### 2.3. LB-GABA Increases Lipolysis in 3T3-L1 Adipocytes

**Figure 4:**
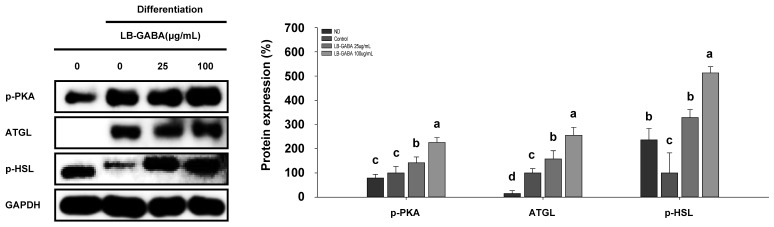
LB-GABA increases lipolysis in 3T3-L1 adipocytes. Adipocytes were cultivated in the presence of a series of concentrations of LB-GABA. The expression of lipolysis proteins (pPKA, ATGL, and pHSL) was determined using Western blot. ND, undifferentiated; control, differentiated. Data are expressed as the mean ± SEM. Values indicated by different letters are significantly different; *p* < 0.05 (a > b > c > d).

The process of lipolysis is characterized by the sequential activity of lipolytic enzymes (ATGL and pHSL), which are induced by PKA activation [35]. Therefore, the present study employed Western blot analysis to determine whether LB-GABA increased lipolysis through the upregulation of the expression of lipolytic enzymes. As shown in Figure 4, Tables S6 and S12, treatment with LB-GABA resulted in a dose-dependent increase in the level of phosphorylation of PKA and the expression levels of its sub-proteins (ATGL and pHSL) in 3T3-L1 cells. This result suggests that LB-GABA increases lipolysis in 3T3-L1 cells.

### 2.4. LB-GABA Activates Energy Metabolism by Promoting the Browning of 3T3-L1 Adipocytes

**Figure 5:**
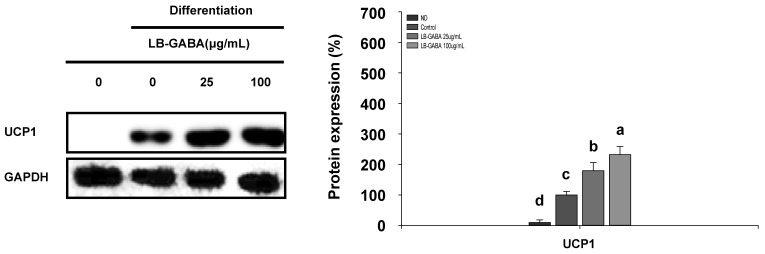
LB-GABA increases browning in 3T3-L1 adipocytes. Adipocytes were cultivated in a series of concentrations of LB-GABA. The expression of a browning protein (UCP1) was determined via Western blot. ND, undifferentiated; control, differentiated. Data are expressed as the mean ± SEM. Values indicated by different letters are significantly different; *p* < 0.05 (a > b > c > d).

White adipocytes can be browned to resemble brown adipocytes [36]. The presence of UCP1 expression is a defining feature of brown adipocytes, which are known to consume energy through the process of inefficient oxidation of FFAs within the mitochondria. Western blot analysis was therefore performed to measure the expression of the brown adipocyte-specific protein UCP1. As shown in Figure 5, Tables S7 and S13, the administration of LB-GABA resulted in a dose-dependent augmentation in the expression of thermogenic proteins. The present research demonstrates that LB-GABA promotes browning by increasing the expression of UCP1 in mitochondria.

### 2.5. Investigation of LB-GABA’s Effect on Reducing Fatty Acid Oxidation in 3T3-L1 Adipocytes

**Figure 6:**
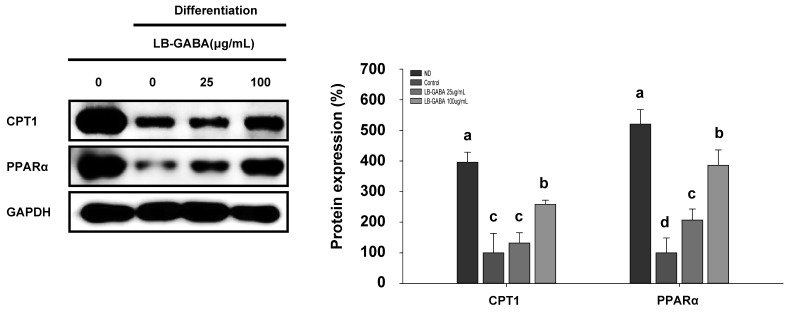
LB-GABA increases fatty acid oxidation in 3T3-L1 adipocytes. Adipocytes were cultured with a series of concentrations of LB-GABA. The expression of fatty acid oxidation proteins (CPT1 and PPARα) was determined via Western blot. ND, undifferentiated; control, differentiated. Data are expressed as the mean ± SEM. Values indicated by different letters are significantly different; *p* < 0.05 (a > b > c > d).

The activities of CPT1 and UCP1 in the mitochondria are linked [37]. The pharmacological ligand of peroxisome proliferator-activated receptor alpha (PPARα) has been shown to activate the expression of genes involved in the processes of fatty acid and glucose oxidation, including carnitine palmitoyltransferase-1A (CPT-1A). At the initial stage of mitochondrial oxidation of long-chain fatty acids, CPT1 functions as a catalyst, facilitating the transfer of long-chain fatty acids from acyl-CoA to carnitine across the mitochondrial membrane [38]. Therefore, we performed a Western blot analysis to determine whether LB-GABA increased the expression of proteins involved in fatty acid oxidation. As shown in Figure 6, Tables S8 and S14, the LB-GABA treatment increased the expression of PPARα and CPT1, which are pivotal in the process of fatty acid oxidation. These findings indicate that LB-GABA enhances the oxidation of fatty acids in 3T3-L1 cells.

## 3. Discussion

Obesity and its associated diseases, including diabetes and hypertension, have emerged as significant global health concerns. In addition, oxidative stress has been demonstrated to induce inflammatory responses, which can result in extensive damage to bodily functions and, in severe cases, even death [39]. Diagnosing and timely managing metabolic syndrome, which is not a disease itself but a risk factor which significantly increases the risk of developing chronic illnesses, can lead to substantial savings—both in terms of time/life quality and financial resources—at the individual and healthcare system levels. Consequently, this study was conducted to ascertain a sustainable method of preventing obesity, a condition that has been demonstrated to diminish the quality of life and reduce life expectancy. The anti-obesity effect of LB-GABA was confirmed in differentiated 3T3-L1 adipocytes.

In this study, we demonstrated that LB-GABA reduces adipogenesis by decreasing the expression of adipogenic proteins, including PPARν and FABP4, in 3T3-L1 adipocytes. These adipogenic factors play important roles after differentiation in white adipocytes. We also showed that LB-GABA can reduce adipogenesis by decreasing the expression of adipogenic factors, an enzyme that is stimulated by TG biosynthesis, and by decreasing PPARγ expression by decreasing LPAATθ expression.

In adipocytes, lipolysis is activated by p-PKA and carried out by enzymes including HSL [40]. In particular, previous studies have shown that the phosphorylation of HSL is induced following PKA activation, releasing FFAs that can be oxidized in the mitochondria [41,42]. The process of lipolysis is defined as the hydrolysis of TGs, leading to the consequent release of fatty acids and glycerol from adipocytes. TG hydrolysis requires the action of lipases such as ATGL and phosphorylated HSL, resulting in the release of free fatty acids. HSL in particular is the key enzyme responsible for the hydrolysis of triglycerides stored in adipose tissue [43]. PKA activation in adipocytes was confirmed, as would be expected to increase lipolysis and mitochondrial activity. Furthermore, it would be expected to increase the expression of ATGL and p-HSL, leading to further hydrolysis to glycerol and free fatty acids. Consistent with this, in this study, LB-GABA treatment effectively increased lipolytic biomarkers, including ATGL and p-HSL, and its downstream factors by increasing the expression of phosphorylated PKA. In summary, LB-GABA not only reduces adipogenesis and lipogenesis but also upregulates lipolytic pathways in 3T3-L1 adipocytes, thereby reducing lipid accumulation.

There has been a significant increase in interest in inducing a switch in differentiation from white adipocytes to BAT-like adipocytes or increasing energy expenditure in BATs. In addition, a current research trend is the use of natural dietary compounds to prevent and treat obesity through lipid metabolism, which is involved in fat browning and energy expenditure [46,47,48,49,50]. Increasing energy expenditure has the potential to prevent metabolic syndrome from obesity, as obesity occurs when energy intake consistently exceeds energy expenditure. A mechanism called ‘browning’ is critically associated with the induction of UCP1 expression in adipocytes [51,52]. Several small molecules, such as berberine and curcumin, appear to induce browning by activating thermogenic transcription factors or modulating key signaling pathways in adipocytes [53,54]. Therefore, a promising therapeutic strategy to increase energy expenditure is the use of substances to stimulate the induction of beige adipocytes or activate brown adipocytes. The process of thermogenesis in BAT-like adipocytes is initiated by the activation of UCP1 [51]. Therefore, this study focused on the differentiation of adipocytes and the inhibition of lipogenesis, as well as the promotion of lipolysis of stored fat through energy consumption and energy metamorphosis. In this study, we found that LB-GABA increased the expression of UCP1, which is an important regulator of this process, thereby inducing the browning of adipocytes and promoting energy metabolism. Furthermore, the results obtained suggest that lipolysis releases FFAs through β-oxidation via PPARa and CPT1, indicating that the β-oxidation of FFAs is also important in brown adipocytes when UCP1 is expressed [52].

In summary, the study utilized 3T3-L1 adipocytes, a model that has been extensively validated for its application in the research domain. The 3T3-L1 model is particularly well suited for investigating the process of adipogenesis, which refers to the transformation of preadipocytes into fully mature adipocytes. This transformation occurs during the developmental stage of mouse embryos. The findings from this study have the potential to inform and guide future experimental research, including the design of animal and clinical trials. Consequently, this cell experimentation confirmed the concentration of LB-GABA administration and its inhibitory effect on obesity. Subsequent investigations will address the anti-obesity impact of LB-GABA in animals. The findings of this study demonstrate that PKA activation by LB-GABA has the potential to enhance metabolic health by inhibiting lipid accumulation and increasing energy expenditure. The positive effects on metabolic health are presumably attributable to lower adiposity, smaller adipocytes, and increased energy expenditure. Consequently, LB-GABA has the potential to serve as a therapeutic agent in the treatment of obesity, with the aim of enhancing metabolic health.

## 4. Materials and Methods

### 4.1. Materials

Dulbecco’s modified Eagle’s medium (DMEM), bovine calf serum (BS), fetal bovine serum (FBS), penicillin streptomycin (P/S), phosphate-buffered saline (PBS), trypsin-EDTA (T/E), and insulin were purchased from Gibco (Gaithersburg, MD, USA). IBMX, dexamethasone, isopropanol, oil red O, phosphatase inhibitors, and the protease inhibitor cocktail were purchased from Sigma-Aldrich (St. Louis, MO, USA). Primary antibodies specific for PPARν (sc7273, 55 kDa), FABP4 (sc-30088, 15 kDa), LPAATθ (sc-68372, 42 kDa), FAS (sc-20140, 270 kDa), PPARα (sc-9000, 55 kDa), p-PKA (sc-136460, 53 kDa), and glyceraldehyde 3-phosphate dehydrogenase (GAPDH, sc365062, 37 kDa) were purchased from Santa Cruz Biotechnology (Dallas, TX, USA). Antibodies specific to ATGL (cs-#1238, 54 kDa) and pHSL (cs-#4139, 81 kDa) were purchased from Cell Signaling Technology (Danvers, MA, USA), and antibodies specific to Lipin 1 (ab70138, 110 kDa), CPT1 (ab128568, 37 kDa), and UCP1 (ab23841, 33 kDa) were purchased from Abcam (Cambridge, UK).

### 4.2. Preparation and Analysis of LB-GABA

LB-GABA production was performed using Lactobacillus brevis fermentation from monosodium L-glutamate. Lactobacillus brevis PF-1, originating from Kimchi, a Republic of Korean fermented vegetable, was cultured in a broth containing monosodium l-glutamate at 27–30 °C for 96–120 h. Culture broth was filter-sterilized, concentrated, and mixed with modified starch. Afterward, it underwent another round of filter sterilization before being subjected to the spray-drying process. LB-GABA comprises approximately 20% GABA (*w*/*w*) and 80% modified food starch [46]. The LB-GABA product contained 323.44 ± 29.48 μg of GABA per milligram (*w*/*w*) in modified food starch (AMOREPACIFIC Co., Seoul, Republic of Korea).

**Figure 7:**
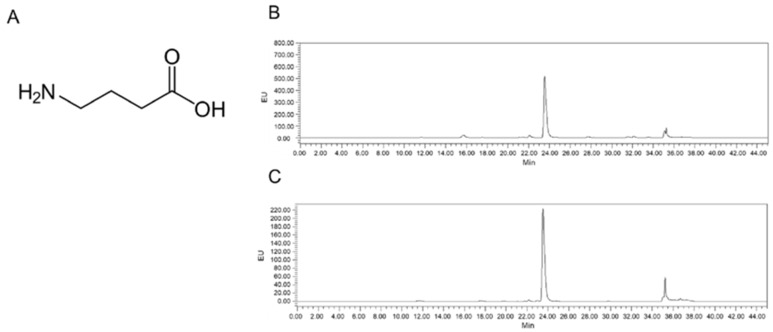
(**A**) Chemical structure and (**B**) high-performance liquid chromatography (HPLC) chromatograms of Lactobacillus-fermented γ-aminobutyric acid (LB-GABA) and the (**C**) GABA standard.

The main peak was detected (Figure 7). The elution time of GABA was 23.95 min (Figure 7B), which was the same as the elution time of synthetic GABA (Figure 7C). Therefore, the LB-GABA sample was confirmed as GABA when compared against the standard synthetic sample [47].

### 4.3. Cell Culture

The 3T3-L1 preadipocytes were purchased from the American Type Culture Collection (Manassas, VA, USA) and maintained in the growth medium (DMEM containing 10% BS, 1% P/S, and 3.7 g/L NaHCO_3_) at 37 °C in a 5% CO_2_ incubator. The 3T3-L1 cells were cultured while replacing the growth medium every 2 days until they became 100% confluent. Two days after they became confluent (D0), the growth medium was replaced with the differentiation medium (DMEM containing 10% FBS, 1% P/S, and 3.7 g/L NaHCO_3_ containing 0.5 mM IBMX, 1 μM dexamethasone, and 4 μg/mL insulin [MDI]) for 2 days. On day 2 (D2), the differentiation medium was removed and replaced with the maintenance medium (DMEM containing 10% FBS, 1% P/S, and 3.7 g/L NaHCO_3_ containing 4 μg/mL insulin) every 2 days up to day 8 (D8).

### 4.4. Cell Viability

The 3T3-L1 preadipocytes were seeded (5 × 10^3^ cells/well) in 96-well plates and incubated overnight in the growth medium. The cells were then treated with LB-GABA (0, 25, 50, 100, or 200 μM) and incubated for a further 24 h. After this, 20 μL of the MTT solution was added to each well, and the cells were incubated for 3 h; then, the MTT-containing medium was removed, and 100 μL of DMSO was added to elute the formazan crystals. The absorbance of each well was then measured at 570 nm (BioTek, Winooski, VT, USA) [48].

### 4.5. Oil Red O Staining

Fully differentiated 3T3-L1 adipocytes were fixed in 10% formaldehyde for 1 h at room temperature and then washed twice with 60% isopropanol. The fixed cells were stained with the oil red O solution (ratio of 6:4 with distilled water) for 20 min at room temperature and then washed with distilled water. After drying, the stained cells were imaged; then, the dye was eluted using 100% isopropanol, and its absorbance was measured at 490 nm.

### 4.6. Western Blotting

The 3T3-L1 adipocytes were washed twice with PBS and then lased in lysis buffer (iNtRON Biotechnology, Seoul, Republic of Korea) containing phosphatase and protease inhibitors. The lysate protein concentrations were determined using a protein assay reagent (Bio-Rad, Hercules, CA, USA). Equal amounts of protein (20 μg) were diluted in 5× sample buffer (50 mM Tris pH 6.8, 2% sodium dodecyl sulfate (SDS), 10% glycerol, 5% β-mercaptoethanol, and 0.1% bromophenol blue) and heated for 5 min at 90 °C. After cooling, the proteins were separated using 8–12% SDS–polyacrylamide gel electrophoresis and transferred to polyvinylidene fluoride membranes. Then, the membranes were blocked with 5% skim milk for 1 h at room temperature. After blocking, the membranes were washed with Tris-buffered saline containing Tween 20 (TBST) and incubated with primary antibodies (1:1000) overnight at 4 °C, followed by incubation with secondary antibodies conjugated with horseradish peroxidase (1:5000) (Santa Cruz Biotechnology) in TBST containing 5% skim milk for 2 h at room temperature [46,47]. Specific protein bands were detected using enhanced chemiluminescence and then imaged using an Amersham Imager 680 (GE Healthcare Life Sciences, Chicago, IL, USA). The localization of proteins was determined using the protein markers of Tri-Glycine 4–20%.

### 4.7. Statistical Analysis

Data are expressed as the mean ± standard deviation (SD). One-way ANOVA with Duncan’s test (SPSS, Chicago, IL, USA) was used to analyze differences among multiple groups. Statistically significant differences were accepted when *p* < 0.05.

All data are expressed as the mean ± standard deviation (SD) and are the results of experiments performed at least three times. Prior to conducting subsequent analyses, normality tests were performed. Differences between groups were analyzed using one-way analysis of variance (ANOVA) with Duncan’s test (SPSS, version 20, Chicago, IL, USA) to identify significant differences.

## 5. Conclusions

The results of this study showed that LB-GABA inhibits adipogenesis and lipogenesis in 3T3-L1, induces lipolysis through FA oxidation, and promotes the browning of adipocytes, confirming the biomarkers of overall obesity through the activation of energy metabolism. In conclusion, LB-GABA exhibits anti-obesity effects within adipocytes. But the long-term use of substances that inhibit adipogenesis may result in a condition called lipodystrophy, which is of genetic or autoimmune origin. It is imperative to be cognizant of the potential issues that may emerge. Further research is required to ascertain the precise anti-obesity effect of LB-GABA in animal models. The present study thus sought to confirm the overall anti-obesity effect of LB-GABA, and it is planned that animal experiments using the established concentration will be conducted in the future. It is hypothesized that this will facilitate the development of new anti-obesity health functional foods.

## Data Availability

All data generated or analyzed during this study are included in this published article.

## Supplementary Materials

The following supporting information can be downloaded at https://www.mdpi.com/article/10.3390/ijms26083554/s1.

## Abbreviations

WAT: white adipose tissue; BAT: brown adipose tissue; LB-GABA: Lactobacillus brevis-fermented gamma-aminobutyric acid; GABA: gamma-aminobutyric acid; PKA: protein kinase A; UCP1: uncoupling protein 1; TG: triglycerides; PPARγ: peroxisome proliferator-activated receptor gamma; FABP4: fatty acid binding protein 4; LPAATθ: lysophosphatidic acid acyltransferase theta; FAS: fatty acid synthase; DGAT1: diacylglycerol acyltransferase 1; FFAs: free fatty acids; CPT1: carnitine palmitoyl transferase 1; ATGL: adipocyte triglyceride lipase; HSL: hormone-sensitive lipase; MGL: monoglyceride lipase; LB-GABA: Lactobacillus-fermented gamma-aminobutyric acid; Lipin1: Phosphatidate phosphatase LPIN1; FA: fatty acid; MTT: 3-(4,5-dimethylthiazol-2yl)-2,5-diphenyltetrazolium bromide; IBMX: 3-Isobutyl-1-methylxanthine; MDI: 0.5 mM 3-isobutyl-1-methylxanthine, 1 μM dexamethasone, and 4 μg/mL insulin; D8: day 8; ND: non-differentiation; PPARα: peroxisome proliferator-activated receptor alpha; CPT-1A: carnitine palmitoyltransferase-1A; DMEM: Dulbecco’s modified Eagle’s medium; BS: bovine calf serum; FBS: fetal bovine serum; P/S: penicillin streptomycin; GAPDH: glyceraldehyde 3-phosphate dehydrogenase; HPLC: high-performance liquid chromatography; DMSO: dimethyl sulfoxide; SDS: sodium dodecyl sulfate; TBST: Tris-buffered saline containing Tween 20; SD: standard deviation; ANOVA: analysis of variance.
