# Supplementary material for: Anti-Obesity Effects of LB-GABA
Source: Int J Mol Sci. 2025 Apr 10;26(8):3554. doi: 10.3390/ijms26083554 (PMC12027266; doi:10.3390/ijms26083554)
Supplement: Supplementary file 1 [file ijms-26-03554-s001.zip › ijms-3476181-supplementary.pdf]

Supplementary data S1

Table S1. One-way ANOVA data of Figure 1.

	SS	Df	MS	F-value	P-value
Between Groups	3015.695	4	753.924	5.412	0.002
Within group	4876.14	35	139.318		
Total	7891.835	39			

Supplementary data S2

Table S2. One-way ANOVA data of Figure 2A.

	SS	df	MS	F-value	P-value
Between Groups	41787.69	3	13929.23	420.47	0
Within group	1457.621	44	33.128		
Total	43245.31	47			

Supplementary data S3

Table S3. One-way ANOVA data of Figure 3A.

PPARγ	SS	df	MS	F-value	P-value
Between Groups	.372	3	.124	88.857	.000
Within group	.011	8	.001		
Total	.384	11			

FABP4	SS	df	MS	F-value	P-value
Between Groups	1.968	3	.656	308.980	.000
Within group	.017	8	.002		
Total	1.985	11			

Supplementary data S4

Table S4. One-way ANOVA data of Figure 3B.

LPAATθ	SS	df	MS	F-value	P-value
Between Groups	1.912	3	.637	86.887	.000
Within group	.059	8	.007		
Total	1.971	11			

Lipin1	SS	df	MS	F-value	P-value
Between Groups	1.411	3	.470	117.946	.000
Within group	.032	8	.001		
Total	1.443	11			

Supplementary data S5

Table S5. One-way ANOVA data of Figure 3C.

FAS	SS	df	MS	F-value	P-value
Between Groups	1.969	3	.656	120.687	.000
Within group	.044	8	.005		
Total	2.012	11			

Supplementary data S6

Table S6. One-way ANOVA data of Figure 4.

pPKA	SS	df	MS	F-value	P-value
Between Groups	1.284	3	.428	26.834	.000
Within group	.128	8	.016		
Total	1.411	11			

ATGL	SS	df	MS	F-value	P-value
Between Groups	1.971	3	.657	46.268	.000
Within group	.114	8	.014		
Total	2.085	11			

pHSL	SS	df	MS	F-value	P-value
Between Groups	2.123	3	.708	33.270	.000

Within group	.170	8	.021		
Total	2.293	11			

Supplementary data S7

Table S7. One-way ANOVA data of Figure 5.

UCP1	SS	df	MS	F-value	P-value
Between Groups	2.383	3	.794	69.844	.000
Within group	.091	8	.011		
Total	2.474	11			

Supplementary data S8

Table S8. One-way ANOVA data of Figure 6.

CPT1	SS	df	MS	F-value	P-value
Between Groups	.927	3	.309	34.104	.000
Within group	.072	8	.009		
Total	.999	11			

PPARα	SS	df	MS	F-value	P-value
Between Groups	.869	3	.290	49.873	.000
Within group	.046	8	.006		
Total	.915	11			

Supplementary data S9

Table S9. Post-Hoc Test data of Figure 3A.

	N	ND	CD	LB-GABA 25	LB-GABA 100
PPARγ	3	83.50±2.57 b	100±2.22 a	66.31±4.82 c	57.81±3.56 d
FABP4	3	22.83±1.66 c	100±2.07 a	94.57±2.20 a	72.66±6.01 b

Supplementary data S10

Table S10. Post-Hoc Test data of Figure 3B.

	N	ND	CD	LB-GABA 25	LB-GABA 100
LPAATθ	3	7.34±6.74 c	100±11.39 a	84.36±4.43 b	72.66±5.87 b
Lipin1	3	5.35±7.00 d	100±9.53 a	57.56±4.11 b	23.39±4.47 c

Supplementary data S11

Table S11. Post-Hoc Test data of Figure 3C.

	N	ND	CD	LB-GABA 25	LB-GABA 100
FAS	3	10.69±10.15 c	100±6.74 a	27.11±6.32 b	2.48±2.89 c

Supplementary data S12

Table S12. Post-Hoc Test data of Figure 4.

	N	ND	CD	LB-GABA 25	LB-GABA 100
pPKA	3	79.17±14.89 c	100±26.38 c	142.11± 23.95 b	225.93± 19.86 a
ATGL	3	15.16±11.64 d	100±17.88 c	157.92± 33.78 b	255.62± 32.43 a
p-HSL	3	236.39± 47.04 b	100±83.11 c	328.69± 32.39 b	512.82± 25.62 a

Supplementary data S13

Table S13. Post-Hoc Test data of Figure 5.

	N	ND	CD	LB-GABA 25	LB-GABA 100

UCP1	3	10.02±8.52 d	100±11.40 c	179.66± 26.67 b	232.28± 26.51 a

Supplementary data S14

Table S14. Post-Hoc Test data of Figure 6.

	N	ND	CD	LB-GABA 25	LB-GABA 100
CPT1	3	396.067± 32.68 a	100±63.50 c	132.12± 33.37 c	258.45± 13.68 b
PPARα	3	520.73± 46.73 a	100±48.94 d	206.72± 36.22 c	385.99± 50.12 b
